# Roles of childhood maltreatment, personality traits, and life stress in the prediction of severe premenstrual symptoms

**DOI:** 10.1186/s13030-022-00240-7

**Published:** 2022-04-28

**Authors:** Chihiro Morishita, Takeshi Inoue, Mina Honyashiki, Miki Ono, Yoshio Iwata, Hajime Tanabe, Ichiro Kusumi, Jiro Masuya

**Affiliations:** 1 Department of Psychiatry, Tokyo Medical University, 6-7-1 Nishishinjuku, Shinjuku-ku, Tokyo, 160-0023 Japan; 2 Faculty of Humanities and Social Sciences, Shizuoka University, 836 Ohya, Suruga-ku, Shizuoka, 422-8529 Japan; 3 Department of Psychiatry, Hokkaido University Graduate School of Medicine, North 15, West 7, Kita-ku, Sapporo, Hokkaido 060-8638 Japan

**Keywords:** Maltreatment, Path analysis, Premenstrual mental symptoms, TCI, Women’s mental health

## Abstract

**Background:**

About 3% to 8% of women of fertile age are thought to have premenstrual dysphoric disorder (PMDD), which is regarded as a serious form of premenstrual syndrome (PMS), although the details of this common condition remain unclear. The aim of this study was to analyze the interrelations of childhood maltreatment, personality traits, and life stress in the etiology of PMS/PMDD.

**Methods:**

A total of 240 adult female volunteers from a community in Japan were investigated, using the following 5 questionnaires: Patient Health Quesstionaire-9, Child Abuse and Trauma Scale, Temperament and Character Inventory (TCI), Life Experiences Survey, and premenstrual dysphoric disorder (PMDD) scale. The questionnaire data were subjected to path analyses to clarify the association between childhood maltreatment and the severity of premenstrual symptoms, mediated by personality traits and life stress.

**Results:**

The 2 path analysis models showed that high harm avoidance (HA) on the TCI and low self-directedness (SD) on the TCI had significant direct effects on the severity of premenstrual symptoms. Moreover, childhood maltreatment was associated with the severity of premenstrual symptoms, both directly and indirectly through personality traits.

**Conclusion:**

Our findings suggest that HA on the TCI might be a risk factor for severe premenstrual symptoms among general women and furthermore that SD on the TCI may be a protective factor. In addition, childhood maltreatment is associated with severe premenstrual symptoms both directly and indirectly through personality traits.

## Background

Premenstrual syndrome (PMS) is a set of somatic and affective symptoms that last for 3 to 10 days before menstruation and promptly decreases or disappears with the start of menstruation [1]. Somatic symptoms include headache, breast tenderness or swelling, abdominal bloating, swelling of the extremities, joint or muscle pain, and weight gain. Affective symptoms include anxiety, depression, angry outbursts, irritability, confusion, and social withdrawal [2]. Several epidemiological studies on PMS have suggested that 20% to 30% of women of reproductive age have this condition [3, 4]. Premenstrual dysphoric disorder (PMDD), designated as a depressive disorder by the Diagnostic and Statistical Manual of Mental Disorders, Fifth Edition (DSM-5) [5], is a serious form of PMS that manifests mainly as affective symptoms. The prevalence of PMDD, estimated from several previous prospective studies, is about 3% to 8% of women of fertile age [6, 7]. Wittchen et al. suggested that the 12-month prevalence of PMDD is 5.8%, based on a prospective survey of 1,488 women in the community aged 14 to 24 years [6]. River-Tovar and Frank also conducted a prospective study of 217 women between the ages of 17 and 29 years over 90 days at a university and reported that 4.6% of them met the diagnostic criteria of late luteal phase dysphoric disorder, which is similar to PMDD [7]. These values are similar to the rates reported in previous studies regarding the prevalence of PMDD in Japanese women [8, 9]. About 4.2% of 861 Japanese female nurses aged 20 to 50 years met the criteria of PMDD according to DSM-IV [10] in a previous interview survey [8]. Moreover, the percentage of women fulfilling the DSM-IV criteria of PMDD [10] was 5.9% in an earlier study of 303 healthy Japanese women ranging from 20 to 45 years of age [9]. On the other hand, some previous studies found that premenstrual symptoms have a negative impact on health-associated quality of life and may lead to decreased occupational productivity and increased health care utilization, and thus pose a potential economic burden [3, 11, 12]. As mentioned above, PMS and PMDD are common conditions and a crucial problem in our society, although information regarding PMS/PMDD is scarce and many women with PMS/PMDD have not been diagnosed or treated. A previous study suggested that approximately 89% of people with PMDD have not been diagnosed [13]. Moreover, an earlier study on Japanese women aged 20 to 49 years suggested that there is a possibility that 1,800,000 women with moderate to severe PMS or PMDD remain untreated [14].

In recent years, various studies have been performed to clarify the etiology and pathophysiology of PMS/PMDD, which might lead to the identification of appropriate treatments. The exact causes of PMS/PMDD remain unclear. However, several reports have suggested that PMS/PMDD might be caused by increased sensitivities to normal hormonal fluctuations and neurotransmitter abnormalities [15, 16]. Moreover, several studies on genetic factors and psychosocial factors as possible risk factors for PMS/PMDD have been conducted. PMDD is grouped under depressive disorders in DSM-5 [5], and shows high comorbidity rates with mood disorders [6, 17–19]. The use of selective serotonin reuptake inhibitors is recommended as the first choice medication in many guidelines [2, 20]. The above lines of evidence suggest that PMS/PMDD share common characteristics with mood disorders, and furthermore, the risk factors for PMS/PMDD might be similar to the risk factors for mood disorders, particularly depression. Several previous studies showed that life stress negatively affects premenstrual conditions [21–23]. Other studies suggested that the experience of maltreatment during childhood, known as a risk factor for depression [24, 25], might also be a risk factor for PMS/PMDD [26, 27]. Furthermore, several studies investigated the personality traits specific to women with PMS/PMDD [8, 28]. At present, the Temperament and Character Inventory (TCI) [29] and the Revised NEO Personality Inventory (NEO-PI-R) [30–32] are often used to assess personality traits in studies on the personality profiles of adults. A few studies have used these personality inventory tests to investigate the personality traits of women with PMS/PMDD. A recent study of 861 nurses reported that higher neuroticism and lower extraversion on the NEO five-factor inventory (NEO-FFI) were found in participants with PMDD than in participants without PMDD, although no significant differences in openness, agreeableness, and conscientiousness on the NEO-FFI were detected between the 2 groups [8]. Hsu et al. compared personality traits on the Tridimensional Personality Questionnaire (TPQ) among women with PMDD, women with major depressive disorder (MDD), and women without these conditions (controls), finding a higher harm avoidance (HA) score in both their PMDD and MDD groups than in their control group, although no significant differences among the 3 groups were detected in novelty seeking (NS) or reward dependence (RD) scores [28]. These results indicated that PMDD and MDD have similar etiologies, although this study had the limitation that it only assessed the temperament dimensions of participants using the TPQ [33], which is the previous edition of the TCI [29] and designed to measure character dimensions as well as temperament dimensions.

As we mentioned above, the 3 factors of childhood maltreatment, personality traits, and life stress have been considered to be possible risk factors for PMS/PMDD, and furthermore they have been assumed to affect each other [34, 35]. However, the interrelations among these factors in the prediction of PMS/PMDD have not been investigated adequately, and therefore, the etiology of PMS/PMDD has remained unclear. On the other hand, the interrelations among these factors in the prediction of depressive symptoms have been investigated by many previous studies, and a theoretical basis for personality traits and life stress as mediators between childhood maltreatment and depressive symptoms has been suggested [36–40]. Some previous studies suggested that personality traits, including neuroticism and affective temperament, might be mediators between childhood maltreatment and adulthood depressive symptoms in the general adult population [36, 37]. Furthermore, other previous studies of the general adult population suggested that childhood maltreatment indirectly increases depressive symptoms through personality traits, which then increases the negative appraisal of stressful life events [38–40]. These previous results suggest that personality traits and life stress might be mediators between childhood maltreatment and depressive symptoms. Therefore, considering that PMS/PMDD share common characteristics with depression, we hypothesized that the experience of maltreatment during childhood and adolescence, personality traits, and recent stressful life events are associated with each other, and furthermore that they influence the premenstrual condition of general women. The definitions of these variables in the present study were as follows. The experience of maltreatment during childhood and adolescence is composed of the following 3 types of negative experiences during childhood and adolescence: punishment (unreasonable and severe punishments), sexual abuse (traumatic sexual experiences and witnessing the sexual mistreatment of other family members), and neglect/negative home atmosphere (physical and emotional neglect, loneliness and lack of attachment, and negative home environment, including parental substance abuse and fighting) [24]. Personality traits are composed of temperaments (traits that are independently heritable, manifest early in life, and involve preconceptual biases in perceptual memory and habit formation), and characteristics (traits that mature in adulthood and influence personal and social effectiveness by insight learning about self-concepts) [29]. Life stress are events during the recent past that have negative impacts on the lives of those who experience them, and furthermore, require the individual to undergo social readjustment [41]. In this study, we aimed to test the hypothesis that the experience of maltreatment during childhood and adolescence, personality traits, and recent stressful life events are associated with each other, and furthermore, influence the premenstrual condition of general women. Therefore, we investigated the associations of the 3 factors of childhood maltreatment, personality traits, and recently experienced stressful life events with premenstrual symptoms, and furthermore we used path analyses to analyze the effects and interactions of these variables on premenstrual symptom severity.

## Methods

### Study design

An observational cross-sectional study was conducted on Japanese adult female volunteers, by collecting data using self-administered questionnaire surveys.

### Participants

The participants of this study were part of a larger study of 1,020 Japanese adult volunteers from the community who were recruited using word of mouth, announcements, and flyers throughout the years of 2014 and 2015. They took part in the present study voluntarily, and received no fee or other compensation. The inclusion criteria were as follows: (a) female; (b) 20 to 45 years of age; and (c) having no serious physical or mental conditions that require frequent doctor’s visits or hospitalizations. Individuals with amenorrhea were excluded. A total of 241 women met the eligibility criteria, accepted participation in our study, and gave written informed consent. The data of 240 women who answered all the items on the questionnaires described below was available for analysis. That of 1 woman who left some of the questions blank was excluded. The women answered the questionnaires and sent the questionnaires to us anonymously. The study protocol was designed in accordance with the Declaration of Helsinki (as revised in Brazil in 2013), and study approval was obtained from the Institutional Review Boards of Tokyo Medical University (approval number T2018-0080) and Hokkaido University (approval number 013-0184).

### Data collection

The data on the participants’ characteristics were collected through a questionnaire that we compiled. It included age, years of education, number of family members, age at first menstruation, marital status, employment status, history of physical diseases and gynecological diseases, treatment history of psychiatric conditions, family history of psychiatric diseases, regularity of menstrual cycle, presence of offspring, use of oral contraceptives/low dose estrogen-progestin, and health-associated behaviors, such as smoking and alcohol use. In addition, the participants were asked to complete the following questionnaires. Accurate information about the participants’ menstrual cycle phases were not obtained, so they would have been at various phases at the time of answering the questionnaires.

### Patient Health Quesstionaire-9 (PHQ-9)

The PHQ-9 is a self-rated questionnaire consisting of 9 items on a 4-point Likert scale, utilized as a screening instrument for major depressive episodes and as an index of depressive symptom severity [42, 43]. The Japanese version of the PHQ-9 was used in this study [44–46]. To assess the severity of depressive symptoms, the total scores of the 9 items were used in the study analyses. In the present study, the Cronbach’s alpha calculated for this scale was 0.893, indicating good internal consistency.

### Child Abuse and Trauma Scale (CATS)

The CATS is a 38-item self-report questionnaire that evaluates the respondent’s present and subjective recognition of the levels of the following 3 types of stress or trauma experienced in their childhood and adolescence: punishment, sexual abuse, and neglect/negative home atmosphere [24]. On each item of the questionnaire, respondents answer the frequencies of their experiences, using a scale of 0 to 4 (0 = never to 4 = always). The Japanese version of the CATS was used in this study [47]. The total score of all items was used in the study analyses. The Cronbach’s alpha calculated for the CATS total score in this study was 0.909.

### Life Experiences Survey (LES)

The LES is a self-rated questionnaire of life changes, consisting of 57 items on a scale of 0 to 7 (-3 = extremely negative to +3 = extremely positive) [40, 41]. Each item is designed to indicate whether respondents viewed episodes that they have experienced in the previous year as positive or negative, and furthermore the degrees of the impacts. A “positive change score” is obtained by adding the impact ratings of episodes perceived as positive, and on the other hand, a “negative change score” is obtained by adding the impact ratings of episodes perceived as negative. The Japanese version of the LES was used in this study. The positive change scores and negative change scores of participants were included in the study analyses. The validity and reliability of the Japanese version of the LES were shown by a previous study of a nonclinical general adult population, as follows [40]. The negative change score significantly and positively correlated with depressive symptoms, state anxiety, and trait anxiety. The positive change score did not correlate with depressive symptoms, state anxiety, or trait anxiety. The test–retest reliability of the LES was confirmed with a moderate intraclass correlation coefficient of 0.47 for the positive change score, and 0.45 for the negative change score, when administered twice within an 8-week period.

### Temperament and Character Inventory (TCI)

The TCI is a self-report questionnaire that assesses the personalities of the respondents. The TCI, which was developed by Cloninger, measures 4 domains of temperament and 3 domains of character [29]. The 4 temperament domains include harm avoidance (HA), reward dependence (RD), novelty seeking (NS), and persistence (P), and the 3 character domains include cooperativeness (C), self-directedness (SD), and self-transcendence (ST). The Japanese 125-item short version with a scale of 1 to 4 (1 = strongly disagree to 4 = strongly agree) was used in this study. The validity and reliability of the Japanese version have been shown in previous studies [48, 49]. The total points of each subscale were included in our analyses. In the present study, Cronbach’s alpha calculated for each subscale of the TCI was as follows: HA, 0.861; RD, 0.642; NS, 0.663; P, 0.532; C, 0.743; SD, 0.860; ST, 0.805.

### Premenstrual dysphoric disorder (PMDD) scale

The PMDD scale was developed by Miyaoka and colleagues [50], according to DSM-IV [10]. The PMDD scale mainly consists of 2 parts. The first part (section A) includes 12 items, assessing the respondent’s PMDD symptom severities on a scale of 1 to 4 (1 = none, 2 = mild, 3 = moderate, 4 = severe). Respondents who answer mild, moderate, or severe to any of the question items are required to answer the second part (section B), consisting of 5 items that measure the degree of influence of the symptoms on school, work, usual social activity, and relationship with others. A respondent is regarded as having PMDD if she answers “severe” for at least 1 of the items regarding mood symptoms in section A, answers “moderate” or “severe” on at least 4 items in section A, and answers severe for at least 1 item in section B. The total points of section A were used in the study analyses. In the present study, Cronbach’s alpha calculated for section A of the PMDD scale was 0.905, indicating excellent internal consistency.

### Statistical analyses

Firstly, the correlations of participants’ characteristics or scores in the above questionnaires with the total points on section A of the PMDD scale were investigated. The Pearson’s product moment correlation coefficient was used to clarify the correlations between the total points on section A of the PMDD scale and continuous variables. The Spearman’s rank correlation coefficient was utilized to demonstrate the correlations between the total points on section A of the PMDD scale and categorical variables. Additionally, correlations between total maltreatment score on the CATS and each personality subscale score on the TCI were assessed using the Pearson’s product moment correlation coefficient. These correlation analyses were performed using SPSS for Windows version 26.0J (IBM., Armonk, NY, USA).

Subsequently, path analysis models were built based on our hypothesis and the correlation coefficients obtained from our above analyses to clarify the effects and interactions of the 3 factors of maltreatment experienced in childhood and adolescence, personality traits, and recent life stress on premenstrual symptoms. PHQ-9 and PMDD scale section A scores were not simultaneously included in the path analysis models, because depressive symptoms assessed by the PHQ-9 overlap considerably with premenstrual symptoms in section A of the PMDD scale. Path analyses using the maximum likelihood estimation were conducted using Mplus version 8.5 software (Muthén & Muthén, Los Angeles, CA, USA), and path coefficients were estimated. Indirect effects for significance were tested by the Sobel test. The Comparative Fit Index (CFI) and Root Mean Square Error of Approximation (RMSEA) were used as indices of the goodness of fit. A CFI greater than 0.95, and a RMSEA less than 0.08 are considered to indicate an acceptable fit; and a CFI greater than 0.97, and a RMSEA less than 0.05 are considered to indicate a good fit [51].

At the beginning of the study, we planned to analyze the effects of about 20 variables on premenstrual symptom severity by multivariable analyses, and calculated the required sample size utilizing G*Power software for Windows version 3.1.9.7 (Heinrich-Heine-Universität Düsseldorf, Düsseldorf, Germany), with an effect size of 0.15, power of 0.95, and significance level (α) of 0.05. The total required sample size was calculated as 222, which indicated that the number of study participants (240 women) in our study was adequate.

For all statistical analyses, a significance level of *p* < 0.05 was used.

## Results

### Demographic characteristics and CATS, TCI, and LES scores of the participants, and correlations with total score on PMDD scale section A

The demographic characteristics, total score on the CATS, the 7 subscores on the TCI, and positive and negative change scores on the LES of the participants (n = 240), as well as their correlations with total score on section A of the PMDD scale are presented in Table 1. According to the criteria of the PMDD scale, which we mentioned above, 10 of the 240 participants (4.2%) were presumed to have PMDD. The PHQ-9 summary score, total score on the CATS, HA score on the TCI, and negative change score on the LES showed significant positive correlations with the total score on section A of the PMDD scale. On the other hand, the SD score on the TCI was significantly negatively correlated with the section A score of the PMDD scale. Younger age and irregular menstrual cycle were associated with a high section A score of the PMDD scale. Several other demographic characteristics were not associated with the section A score of the PMDD scale. TCI subscores, except for HA and SD (namely, NS, RD, P, C, and ST scores), and the positive change score on the LES were not correlated with the section A score of the PMDD scale.

**Table 1:** Demographic characteristics and questionnaire scores of the participants

			Number or mean (S.D.)	Correlation with PMDD scale section A score
Demographic characteristic				
Age (years)			32.16 (6.82)	*r* = –0.18**
Education (years)			14.90 (1.66)	*r* = 0.02 (n.s.)
Number of family members			1.53 (1.59)	*r* = –0.01 (n.s.)
Age of first menstruation (years)			12.38 (1.41)	*r* = 0.04 (n.s.)
Marital status (married: single)			102: 138	*ρ* = –0.08 (n.s.)
Employment status (employed: nonemployed)			203: 31	*ρ* = –0.04 (n.s.)
History of physical diseases (yes: no)			32: 208	*ρ* = 0.08 (n.s.)
History of gynecological diseases (yes: no)			64: 175	*ρ* = –0.01 (n.s.)
Treatment history of psychiatric conditions (yes: no)			18: 222	*ρ* = 0.01 (n.s.)
Family history of psychiatric diseases (yes: no)			16: 223	*ρ* = –0.06 (n.s.)
Menstrual cycle (irregular: regular)			59: 175	*ρ* = 0.13*
Offspring (yes: no)			79: 159	*ρ* = –0.12 (n.s.)
OCs / LEP (use: non-use)			15: 225	*ρ* = 0.10 (n.s.)
Smoking habit (yes: no)			23: 217	*ρ* = –0.02 (n.s.)
Drinking habit (yes: no)			40: 198	*ρ* = 0.06 (n.s.)
Questionnaire score				
PHQ-9			4.72 (5.07)	*r* = 0.40**
CATS total			26.95 (17.02)	*r* = 0.26**
LES	Positive		0.75 (1.90)	*r* = 0.09 (n.s.)
	Negative		0.98 (2.59)	*r* = 0.20**
TCI	Temperament	Novelty seeking (NS)	29.32 (5.91)	*r* = –0.004 (n.s.)
		Harm avoidance (HA)	34.42 (8.25)	*r* = 0.29**
		Reward dependence (RD)	28.34 (4.80)	*r* = 0.03 (n.s.)
		Persistence (P)	7.50 (2.20)	*r* = 0.04 (n.s.)
	Character	Self-directedness (SD)	44.43 (9.77)	*r* = –0.27**
		Cooperativeness (C)	47.54 (6.82)	*r* = –0.05 (n.s.)
		Self-transcendence (ST)	11.23 (5.76)	*r* = 0.11 (n.s.)
	PMDD scale section A		22.31 (7.68)	

* *p* < 0.05, ** *p* < 0.01

*r*, Pearson’s product moment correlation coefficient; *ρ*, Spearman’s rank correlation coefficient; *CATS* Child Abuse and Trauma Scale, *LEP* low dose estrogen-progestin, *LES* Life Experiences Survey, *n.s.* not significant (*p* ≥ 0.05), *OCs* oral contraceptives, *PHQ-9* Patient Health Quesstionaire-9, *PMDD scale* premenstrual dysphoric disorder scale, *S.D.* standard deviation, *TCI* Temperament and Character Inventory

### Correlations between total maltreatment score on the CATS and personality dimension scores on the TCI

Correlations between the total maltreatment score on the CATS and each personality subscale score on the TCI are shown in Table 2. Statistically significant correlations of HA, RD, SD, C, and ST scores on the TCI with the total score on the CATS were detected. The HA score on the TCI showed a weak positive correlation with the total score on the CATS, and the ST score on the TCI showed a very weak positive correlation with the CATS total score. On the other hand, the SD score on the TCI moderately was negatively correlated with the total score on the CATS, and the RD and C scores on the TCI were weakly, negatively correlated with the CATS total score.

**Table 2:** Correlations between maltreatment score on CATS and personality dimension scores on TCI

	CATS total	NS^a^	HA^a^	RD^a^	P^a^	SD^a^	C^a^	ST^a^
CATS total	1.00	–0.01	0.25^**^	–0.24^**^	–0.09	–0.46^**^	–0.23^**^	0.14^*^
NS^a^	–	1.00	–0.43^**^	–0.02	–0.01	0.02	–0.08	0.25^**^
HA^a^	–	–	1.00	0.03	–0.08	–0.53^**^	–0.08	–0.14^*^
RD^a^	–	–	–	1.00	0.17^**^	0.33^**^	0.45^**^	–0.11
P^a^	–	–	–	–	1.00	0.07	0.17^**^	0.18^**^
SD^a^	–	–	–	–	–	1.00	0.30^**^	–0.27^**^
C^a^	–	–	–	–	–	–	1.00	0.02
ST^a^	–	–	–	–	–	–	–	1.00

Values represent *r*-values

^a^NS, HA, RD, P, SD, C, and ST were assessed by the TCI

* *p* < 0.05, ** *p* < 0.01

Values (*r*) without * or ** did not reach statistical significance (*p* < 0.05)

*r*, Pearson’s product moment correlation coefficient; CATS, Child Abuse and Trauma Scale; TCI, Temperament and Character Inventory; NS, novelty seeking; HA, harm avoidance; RD, reward dependence; P, persistence; SD, self-directedness; C, cooperativeness; ST, self-transcendence

### Effects and interactions of childhood maltreatment, personality traits, and life stress on premenstrual symptom severity (path analysis models)

We built path analysis models based on our hypothesis and the results of correlation analyses, to clarify the interrelations among childhood maltreatment, personality traits, recent life stress, and premenstrual symptom severity. We initially built a path analysis model that incorporated both HA and SD scores measured by the TCI, in addition to total score on the CATS, negative change score assessed by the LES, premenstrual symptom severity evaluated by the PMDD scale, and the demographic characteristics that significantly correlated with premenstrual symptom severity (namely, age and menstrual cycle). However, the goodness of fit for this model did not indicate an acceptable fit. Therefore, we performed the following 2 path analyses, treating HA and SD separately, as exploratory analyses.

**Fig. 1:**
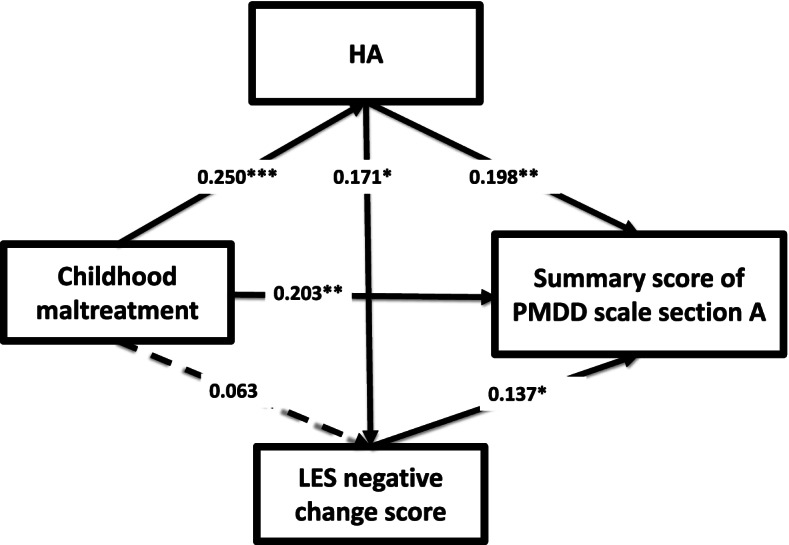
Results of path analysis incorporating the HA score on the TCI. In addition to the HA score on the TCI, the total score on the CATS, the negative change score on the LES, premenstrual symptom severity evaluated by the PMDD scale, and control variables (age and menstrual cycle) were incorporated in the path analysis of 240 adult female volunteers from the Japanese community. Control variables are not shown. Solid arrows indicate statistically significant direct effects. Broken arrows indicate statistically nonsignificant direct effects. Numbers beside the single-headed arrows are standardized path coefficients. * *p* < 0.05, ** *p* < 0.01, and *** *p* < 0.001. CATS, Child Abuse and Trauma Scale; HA, harm avoidance; LES, Life Experiences Survey; PMDD scale, premenstrual dysphoric disorder scale; TCI, Temperament and Character Inventory

The results of the path analysis that incorporated the HA score on the TCI, the total score on the CATS, the negative change score assessed by the LES, and premenstrual symptom severity evaluated by the PMDD scale, in addition to control variables (age and menstrual cycle), are shown in Fig. 1. The goodness of fit was a CFI of 1.000 and a RMSEA of 0.000, which indicate a good fit. The estimated *R*-squared value for premenstrual symptom severity in this model was 0.171. A high HA score on the TCI, a high total score on the CATS, and a high negative change score on the LES had significant direct effects on premenstrual symptom severity. Moreover, the indirect effect of the total CATS score on the severity of premenstrual symptoms through HA was statistically significant (β = 0.049, *p* = 0.012), although the indirect effect of the total CATS score on the severity of premenstrual symptoms through the LES negative change score was not statistically significant. Furthermore, the indirect effect of HA on premenstrual symptom severity through negative life experiences was not statistically significant.

**Fig. 2:**
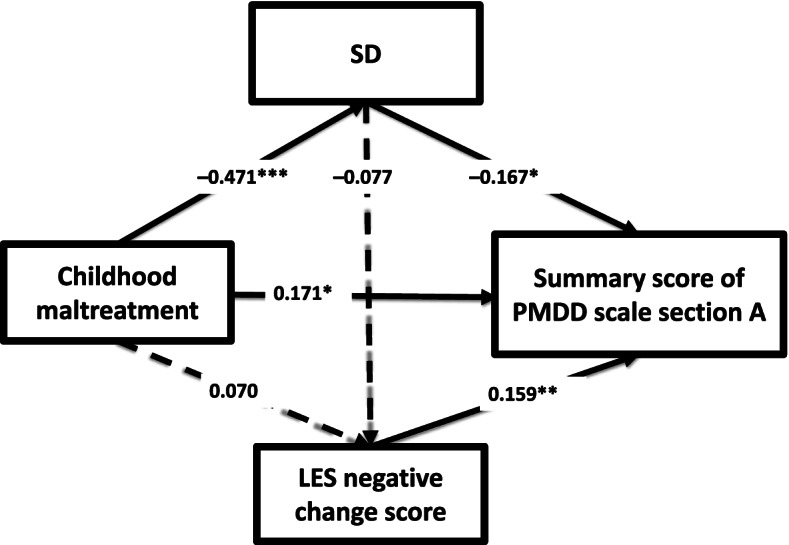
Results of path analysis incorporating the SD score on the TCI. In addition to the SD score on the TCI, the total score on the CATS, the negative change score on the LES, premenstrual symptom severity evaluated by the PMDD scale, and control variables (age and menstrual cycle) were incorporated in the path analysis of 240 adult female volunteers from the Japanese community. Control variables are not shown. Solid arrows indicate statistically significant direct effects. Broken arrows indicate statistically nonsignificant direct effects. Numbers beside the single-headed arrows are standardized path coefficients. * *p* < 0.05, ** *p* < 0.01, and *** *p* < 0.001. CATS, Child Abuse and Trauma Scale; SD, self-directedness; LES, Life Experiences Survey; PMDD scale, premenstrual dysphoric disorder scale; TCI, Temperament and Character Inventory

We built a path analysis model including SD from the TCI instead of HA. Namely, the path analysis model incorporated the SD score on the TCI, the total score on the CATS, the negative change score assessed by the LES, and the total points of section A of the PMDD scale, in addition to control variables (age and menstrual cycle) (Fig. 2). The goodness of fit was a CFI of 1.000 and a RMSEA of 0.000, which indicate a good fit. The estimated *R*-squared value for premenstrual symptom severity in this model was 0.157. A low SD score on the TCI, a high total score on the CATS, and a high negative change score on the LES had significant direct effects on the total section A score on the PMDD scale. Moreover, the indirect effect of the total CATS score on premenstrual symptoms through SD was statistically significant (β = 0.079, *p* = 0.019), although the indirect effect of the total CATS score on premenstrual symptoms through the LES negative change score was not statistically significant. Furthermore, the indirect effect of SD on premenstrual symptoms through negative life experiences was not statistically significant.

In addition to the above 2 models that included HA and SD, we built path analysis models including the other temperament and character dimensions (namely, NS, RD, P, C, and ST), although these temperament and character dimensions did not statistically correlate with premenstrual symptoms, and furthermore did not strongly or moderately correlate with the total score on the CATS (Tables 1 and 2). These models (namely, path analysis models including NS, RD, P, C, and ST) showed that both the direct effect of each personality trait on premenstrual symptoms and the indirect effect of childhood maltreatment on premenstrual symptoms through each personality trait were not statistically significant.

## Discussion

### Summary of our results

The main findings of this study are as follows. The 2 path analysis models showed that a high HA score on the TCI and a high negative change score on the LES have direct effects on premenstrual symptom severity, and furthermore that a low SD score on the TCI has a direct effect on premenstrual symptom severity. Therefore, we considered that HA and recent negative life experiences might be risk factors for severe premenstrual symptoms and furthermore that SD may have a protective role against premenstrual symptoms. Moreover, the direct and indirect effects of the CATS score on the section A score of the PMDD scale indicated that childhood maltreatment is directly associated with the severity of premenstrual symptoms and indirectly through personality traits. These findings support our hypothesis that childhood maltreatment, personality traits, and life stress interact with each other in the etiology of PMS/PMDD.

### Comparison with previous studies

We investigated the associations between premenstrual symptom severity and personality traits assessed by the TCI, which is designed to measure character and temperament dimensions [29, 33]. The results of this study confirmed that HA, which is the temperament dimension that has been hypothesized to be associated with serotonergic function and corresponds to behavioral inhibition might be a risk factor for premenstrual symptoms. Furthermore, our results suggested that SD, which is the character dimension that refers to the ability to control individual behavior to match the situation in concordance with an individual’s goals and values, might play a protective role against premenstrual symptoms. This new finding of the role of character dimensions of the TCI in premenstrual symptoms might be useful towards clarifying the etiology of PMS/PMDD, and therefore this is considered to be a strength of our study. Regarding the temperament dimensions of the TCI, HA was considered to be a risk factor for premenstrual symptoms, which was similar to the results shown in a few previous studies using the TPQ, the previous edition of the TCI [28, 52, 53]. As we mentioned in the Introduction section, Hsu et al. reported that the HA score on the TPQ tended to be higher in women with PMDD and MDD than in women without these conditions [28]. Furthermore, Eissa et al. identified a statistically significant correlation between the severity of premenstrual symptoms and the HA score, but not NS and RD scores [52]. Freeman et al. emphasized that the HA dimension scores of PMS women seeking medical treatment were higher than those of general women, but lower than those of women with MDD and women with premenstrual exacerbation of MDD [53]. The personality data of the people analyzed in these previous studies that used the TPQ did not include character dimensions, and furthermore the statistical powers of these studies were weak, because of the small sample sizes. Our path analyses results indicate a robust association between HA and premenstrual symptoms. Regarding the association between personality traits assessed by the TCI and premenstrual symptoms, to our knowledge there has only been 1 previous study by Telek et al., which found that healthy women with luteal phase symptom worsening had significantly higher NS, SD, C, and ST scores and a lower HA score than healthy women without luteal phase symptom worsening [54]. The results of this previous study appeared to show opposite trends to the results of our present study and previous studies using TPQ [28, 52, 53]. This discrepancy might be owing to differences in the participants of these studies. In the study by Telek et al., they aimed to identify the personality traits that protect women who experience distressing fluctuations of symptoms parallel to their menstrual cycle from developing PMS and PMDD. They hence focused on 40 healthy women after excluding women with any psychiatric disorders and menstrual cycle-associated disorders, such as PMDD and PMS, and compared personality dimensions on the TCI between a group of healthy women with luteal phase symptom worsening and a group of healthy women without luteal phase symptom worsening. On the other hand, our study, as well as previous studies using TPQ [28, 52, 53], aimed to identify the personality traits of women who experience PMS and PMDD and focused on a population that included women with PMS and PMDD. However, we considered that further studies comparing personality traits on the TCI of women who develop PMS and PMDD with women who do not when experiencing luteal phase symptom worsening are needed to clarify the difference between our present study and the previous study by Telek et al. On the other hand, many previous studies investigated the associations between depressive symptoms and personality traits evaluated by the TCI and suggested that lower SD and higher HA scores were likely to be observed in people with depressive conditions [55, 56]. When these findings of depressive symptoms and our findings of premenstrual symptoms are combined, low SD and high HA are considered to be common personality traits that are shared by those with MDD and PMS/PMDD, and furthermore, these similarities in personality profiles might reflect the common etiology of PMS/PMDD and MDD.

Additionally, we found that the experience of maltreatment in childhood might exacerbate premenstrual symptoms. As we mentioned in the Introduction section, similar results were shown in previous studies [26, 27]. Soydas et al. compared the childhood abuse of 70 patients with PMDD and 78 healthy controls, using a questionnaire assessing 3 types of childhood trauma experiences (physical abuse, sexual abuse, and emotional abuse/emotional neglect). They showed that patients with PMDD had significantly higher scores than healthy controls in all 3 subscale scores and total scores, although the effects of the other factors, including life stress, were not considered [27]. Moreover, Bertone-Johnson et al. suggested in a prospective cohort study, that early life abuse was significantly associated with increased risk of PMS in middle to late reproductive age women [26]. Given the results of our study and previous studies, childhood maltreatment can be considered to be a risk factor for PMS/PMDD, similar to childhood maltreatment being a risk factor for MDD [24, 25].

The 2 path analysis models demonstrated that the negative change score on the LES had a direct effect on the total section A score of the PMDD scale, which suggested that recent negative life experiences might tend to trigger premenstrual symptoms, as has been suggested by many previous studies [23, 57]. On the other hand, interestingly, the indirect effects of personality traits and childhood maltreatment on premenstrual symptom severity through negative life experiences were not significant, and therefore we did not identify negative life experiences as a significant mediator.

As we mentioned above, it is valuable to understand each association between premenstrual conditions and childhood maltreatment, personality traits, and recently experienced life events. The greatest strength of our study is that we addressed the interrelations of these factors regarding their association with PMS/PMDD, and we identified HA and SD as statistically significant mediators between childhood maltreatment and premenstrual symptom severity. To our knowledge, this is the first study to suggest that personality traits measured by the TCI might act as mediators between the experience of childhood maltreatment and premenstrual symptom severity. Our previous study also investigated the interrelations of some risk factors regarding their association with PMS/PMDD, including personality traits evaluated by the Temperament Evaluation of Memphis, Pisa, Paris and San Diego-autoquestionnaire version (TEMPS-A) instead of the TCI [58–60]: specific affective temperaments were identified as mediators [61]. However, the TEMPS-A was established to evaluate a patient’s personality dysregulation in the premorbid stages of affective disorders [60], and therefore it may not be appropriate for people without these conditions. On the other hand, the TCI is frequently used to assess general adult personality traits [29]. Therefore, the results of our study suggest that HA and SD on the TCI are mediators between childhood maltreatment and future premenstrual symptoms in general non-clinical women and that these personality traits are key factors that need to be addressed by clinicians.

On the other hand, we assume that other factors, including biological factors, in addition to variables identified as being statistically associated with the severity of premenstrual symptoms in this study, might influence premenstrual symptom severity [16, 62]. In the future, the association of various biological factors and other psychosocial factors with premenstrual symptom severity should be evaluated, which is expected to lead to clarification of the etiology of PMS/PMDD. Furthermore, whether interventions targeting the factors associated with premenstrual symptom severity (e.g., parent training and stress management training) ameliorate premenstrual symptoms and prevent PMS/PMDD should be analyzed in the future, which might lead to the identification of appropriate treatments.

### Limitations of our study

This study has several limitations. First, the premenstrual symptoms of the participants may not have been assessed accurately. This is because the PMDD scale, which is a self-assessment questionnaire, was administered only once, retrospectively, and diagnostic interviews were not conducted. Additionally, although the menstrual cycle phase might affect the PMDD scale scores, information about the participants’ menstrual cycle phase at the time of answering the PMDD scale was not obtained and the influence of the menstrual cycle phase was not considered in the study analyses. Furthermore, childhood maltreatment was evaluated retrospectively using a self-administered questionnaire, and hence there is the possibility of bias in the evaluation of the participants’ experience of childhood maltreatment. Regarding participants’ personality traits, because the Cronbach’s alpha coefficients for RD, NS, and P measured by the TCI were all less than 0.70 in the present study, their reliabilities might be limited. Secondly, the estimated *R*-squared value for premenstrual symptom severity in our models was not high, which might suggest that other factors, including steroid hormones and other psychosocial factors, are associated with the severity of premenstrual symptoms. Further evaluation of the effects of biological factors on the risk of PMS/PMDD are needed in the future. Thirdly, the study participants were adult female volunteers from a single community in Japan, and therefore it remains unclear whether the findings can also be applied to other groups of patients with PMS/PMDD. Finally, the design was cross-sectional, and therefore variables identified as factors statistically associated with the severity of premenstrual symptoms might not necessarily be useful for predicting PMS/PMDD.

## Conclusions

Our 2 path analysis models demonstrated the interrelations of childhood maltreatment, personality traits, and life stress in the etiology of PMS/PMDD. These findings suggest that HA of the TCI might be a risk factor for PMS/PMDD and furthermore that SD of the TCI might play a protective role against the development of PMS/PMDD. Furthermore, we demonstrated a direct effect of childhood maltreatment on severe premenstrual symptoms and an indirect effect mediated by personality traits.

## Data Availability

The datasets used and analyzed during the current study are available from the corresponding author on reasonable request.

## Abbreviations

C: cooperativeness
CATS: Child Abuse and Trauma Scale
DSM-5: Diagnostic and Statistical Manual of Mental Disorders, Fifth Edition
LES: Life Experiences Survey
HA: harm avoidance
MDD: major depressive disorder
NEO-FFI: NEO five-factor inventory
NEO-PI-R: Revised NEO Personality Inventory
NS: novelty seeking
P: persistence
PHQ-9: Patient Health Quesstionaire-9
PMDD: premenstrual dysphoric disorder
PMDD scale: premenstrual dysphoric disorder scale
PMS: premenstrual syndrome
RD: reward dependence
SD: self-directedness
ST: self-transcendence
TCI: Temperament and Character Inventory
TEMPS-A: Temperament Evaluation of Memphis, Pisa, Paris and San Diego-autoquestionnaire version
TPQ: Tridimensional Personality Questionnaire
